# Manual Video-Based Skill Assessment Does Not Predict Postoperative Outcomes in Complex Robotic Upper Gastrointestinal (UGI) and Hepatopancreatobiliary (HPB) Surgery

**DOI:** 10.7759/cureus.114110

**Published:** 2026-08-07

**Authors:** Nicholas Favazza, Yossef Rubinov, Alexis Benjamin, Xhesika Shanja, Sandeep Anantha, Anthony Antonacci, Gary Deutsch

**Affiliations:** 1 Department of Internal Medicine, Tufts Medical Center, Boston, USA; 2 Department of Radiology, Montefiore Medical Center, Albert Einstein College of Medicine, New York City, USA; 3 Department of Obstetrics and Gynecology, South Shore University Hospital, Bay Shore, USA; 4 Department of Surgery, Icahn School of Medicine at Mount Sinai, New York City, USA; 5 Department of Surgical Oncology, Long Island Jewish Medical Center, New Hyde Park, USA; 6 Department of Surgical Oncology, North Shore University Hospital, Manhasset, USA; 7 Department of Surgical Oncology, New York University (NYU) Langone Hospital, Mineola, USA

**Keywords:** assessment, complexity, gears, procedure, robotics, skill

## Abstract

Background: The relationship between technical skill, measured through "gold standard" manual video-based review, and patient outcomes has not been clearly defined in complex robotic upper gastrointestinal (UGI) or hepatopancreatobiliary (HPB) surgery.

Methods: Global Evaluative Assessment of Robotic Skills (GEARS) scores were prospectively captured between 2015 and 2020 across four tertiary-care institutions. Clinical, operative, and postoperative outcome data were retrospectively reviewed for all UGI (non-bariatric) and HPB surgery cases with available GEARS scores (range: 5-25). Subgroup analyses were also performed based on GEARS components and procedure length. This study has received approval from the institutional review board (IRB).

Results: Of 4,673 cases evaluated across four tertiary-care institutions, 164 patients had UGI and HPB surgeries with evaluable GEARS; 95 UGI (esophagus, n=19; gastric, n=76) and 69 HPB (pancreas, n=22; hepatobiliary, n=47) were included. Mean age was 62.8±13.7 years; 41.5% and 56.7% identified as White and male, respectively. A total of 44 patients received neoadjuvant therapy. Mean procedure length and overall GEARS were 272.5±125.8 min and 19.9±0.86 for all procedures. Procedure length and GEARS scores demonstrated a weak inverse correlation (r = -0.275, p≤0.001). Postoperative complication rate was 51.8% (12.2% Clavien-Dindo classification of surgical complications {C-D} grade 3+) for all procedures. In multivariable analysis, each additional minute of procedure length was associated with increased odds of postoperative complications (OR=1.007, p<0.001), whereas GEARS score was not (OR=1.218, p=0.348). Findings remained consistent independent of surgery type, GEARS component score, or procedure length cohort.

Conclusion: Procedure length is inversely associated with technical skill, as measured by GEARS. However, GEARS is not directly associated with postoperative outcomes in robotic UGI and HPB surgery. Future efforts focused on performance assessment may increase accuracy and utility by incorporating the entirety and complexity of an operation.

## Introduction

Operative technical performance directly impacts surgical outcomes, with wide variation in technical skill existing among practicing surgeons and accounting for more than 25% of the discrepancy in outcome [1]. Furthermore, as minimally invasive surgery becomes more widespread, there is a pressing need to quantify this relationship in robotic surgery. This applies to both the expert surgeon, who must consistently reflect on accurate and objective measures of their performance to continuously improve their skill set, as well as the novice whose technical skill must be accurately and objectively assessed by their more experienced colleagues to determine their progress along the surgical learning curve.

In complex robotic operations, such as gastrointestinal and hepatopancreatobiliary (HPB) surgery, the quality of surgical technique may be even more critical. Current literature has suggested that the learning curve for robotic-assisted pancreaticoduodenectomy for those without formal robotic training may be up to 80 cases based on improvements in several outcome measures, including estimated blood loss, conversion rate, postoperative pancreatic fistula formation, readmission, and total operating room time [2]. Similarly, the learning curve for robotic-assisted hepatectomy has been estimated at approximately 40 cases [2]. This pattern of increased operative experience leading to better outcomes has also held true for robotic-assisted gastric resections [3]. Particularly interesting is the relationship between decreased operative time across procedures and outcomes, as some literature has suggested that increased operative times in laparoscopic procedures may correlate with increased rates of complications [4]. With these factors in mind, both robotic mentorship programs and training curricula have been implemented at several institutions and have been shown to improve trainees’ initial performance [2,3]. One well-established program developed a five-step, mastery-based training program to allow trainees to achieve proficiency in complex surgical oncology and HPB surgery. Review of this program revealed significant improvements in trainee performance in a simulated surgical environment as measured by decreases in trainee time to perform surgical tasks, decreased error rate, and increased technical proficiency scores in simulations mimicking the creation of gastrojejunostomy, hepaticojejunostomy, and pancreaticojejunostomy. Preliminary work in evaluation of trainee performance has been performed primarily via review of video files of robotic procedures [3].

While some institutions may have formal training and established evaluation procedures regarding complex robotic surgery, the Global Evaluative Assessment of Robotic Skills (GEARS) score is currently the standard method for assessing the quality of technical skill in robotic surgery. The GEARS score is designed to provide the individual surgeon with quantitative feedback in several domains of robotic surgery, including depth perception, bimanual dexterity, efficiency, force sensitivity, and robotic control. Procedure videos are uploaded to the platform, and short key segments are then analyzed and scored by expert surgeons. Each domain is scored on a Likert scale between 1 and 5, and the overall GEARS score attained can range between 5 and 25, with higher scores signifying better surgical technique [5,6].

There is limited literature demonstrating a relationship between manual video-based review and postoperative outcomes in gastrointestinal and HPB surgery. The few existing studies suggest that technical scoring of video reviews may be useful in predicting patient outcomes in specific settings, including robotic-assisted pancreatojejunostomy and radical prostatectomy [7,8]. With this in mind, we set out to define the relationship between GEARS score and the occurrence of postoperative complications in robot-assisted upper gastrointestinal (non-bariatric) and hepatopancreatobiliary surgical oncology procedures. We hypothesized that higher GEARS scores would be associated with lower odds of postoperative complications.

## Materials and methods

A retrospective review of 4,673 robotic cases at four tertiary care institutions from 2015 to 2020 was performed. All eligible upper gastrointestinal (non-bariatric) and hepatopancreatobiliary robotic procedures with available GEARS assessments were included in the present analysis, regardless of procedural complexity. Expert raters were recruited through established academic and professional networks based on their expertise in robotic surgery. Eligible reviewers were experienced robotic surgeons with substantial procedural experience and familiarity with objective surgical skill assessment. Before formal evaluation, all raters completed standardized training and calibration on the Global Evaluative Assessment of Robotic Skills (GEARS) instrument to promote consistent interpretation of the scoring criteria. Surgical videos were de-identified and presented in random order, and reviewers independently scored each video using the validated GEARS domains (depth perception, bimanual dexterity, efficiency, force sensitivity, and robotic control). Each video was assessed by at least two blinded expert raters, and the resulting scores were aggregated to reduce individual reviewer bias and improve the reliability of the reference standard.

Data were obtained from the institutional electronic medical record of all cases with available GEARS scores who underwent upper gastrointestinal (UGI) (non-bariatric) and HPB robotic surgeries. The overall GEARS score, as well as the individual subcomponent scores, were collected, along with several other variables thought to influence individual procedure outcomes. These included age at the time of surgery, sex, overall procedure length, whether the patient received neoadjuvant chemotherapy, body mass index (BMI), history of prior surgery, and tumor, node, metastasis (TNM) stage of malignancy [4,9-12]. Pathologic stage was classified according to the American Joint Committee on Cancer (AJCC) eighth edition. The frequency and specific nature of postoperative complications were also recorded, defined as both surgical (wound infection, bleeding requiring blood transfusion, and abscess requiring drainage or reoperation) and non-surgical (pneumonia, readmission within 30 days of operation, emergency department visit within 30 days of operation, and unplanned reoperation within 30 days of the initial operation) [13]. Complication data were also categorized by severity using the Clavien-Dindo classification of surgical complications (C-D) scale, with complications rated ≥3 considered most severe [14].

The variables were evaluated with Pearson's correlation coefficient to assess for any type of relationship. Data were then analyzed via several multiple logistic regression analyses using SPSS Statistics version 29.0.1.0 software (Armonk, NY: IBM Corp.), with the primary dependent endpoint defined as the occurrence of at least one grade 3+ postoperative complication. The study population was then subdivided into long procedures and short procedures. Long procedures were defined as those procedures that exceeded the overall median procedure length for the complete study population. The mean GEARS score, age, and BMI of these subgroups were compared using the t-test. Each subgroup was then further analyzed using multiple logistic regression analysis as described previously.

## Results

A total of 164 patients underwent UGI or HPB surgeries within the department of surgical oncology. Descriptive and clinical variables were compiled for all patients (Table 1). The pathologic stage was well distributed in the study population as follows: stage I (n=45) 27.4 %, stage II (n=29) 17.7%, stage III (n=23) 14%, and stage IV (n=4) 2.4%. The total number of patients who experienced at least one postoperative complication, as defined by the criteria outlined in the methods section, was 85 (51.8% total, 12.2% C-D grade 3+).

**Table 1:** Patient demographics divided into short, long, and overall procedure. UGI: upper gastrointestinal; HPB: hepatopancreatobiliary; GEARS: Global Evaluative Assessment of Robotic Skills

Variables	All procedures	Short procedures	Long procedures
Mean overall GEARS score	19.9±0.86	20.2±0.7	19.7±0.94
Mean procedure length (min)	272.5±125.8	169.1±57.6	373.4±86
Mean age at time of surgery (years)	62.8±13.7	61.6±15.3	64±11.9
Mean BMI at time of surgery (kg/m^2^)	28±6.5	28.8±7.0	27.2±5.9
Neoadjuvant therapy, n (%)	44 (26.8)	9 (11.1)	35 (42.2)
Past surgical history, n (%)	124 (75.6)	58 (71.6)	66 (79.5)
Sex			
Male, n (%)	93 (56.7)	42 (51.9)	51 (61.4)
Female, n (%)	71 (43.3)	39 (48.1)	32 (38.6)
Race			
White, n (%)	64 (39)	30 (37)	34 (41)
Asian, n (%)	46 (28)	16 (19.8)	30 (36.1)
African American, n (%)	20 (12.2)	12 (14.8)	8 (9.6)
Multiracial/other, n (%)	31 (18.9)	21 (25.9)	10 (12)
Unknown, n (%)	3 (1.8)	2 (2.5)	1 (1.2)
Procedure type			
UGI, n (%)	95 (57.9)	38 (46.9)	57 (68.7)
HPB, n (%)	69 (42.1)	43 (53.1)	26 (31.3)
Total subjects, n	164	81	83

Age, BMI, administration of neoadjuvant chemotherapy, history of prior surgery, and TNM stage were not identified as significant in predicting the likelihood of postoperative complications after robotic-assisted UGI and HPB surgeries. Procedure length was the only independent variable that showed statistical significance in multivariate analysis, with increased procedure lengths conferring increased odds of postoperative complications (OR=1.007, p<0.001) (Table 2). Although Pearson correlation revealed an inverse correlation between procedure length and overall GEARS score (r = -0.275, p<0.001), the overall GEARS score was not a statistically significant predictor of postoperative complications (OR=1.218, p=0.348). The model was significant (X^2^=27.054, p=0.005), with the model accounting for 20.3% of the variance (Nagelkerke R^2^).

**Table 2:** Multiple logistic regression analysis relating several covariates to postoperative complication odds for the complete study population. Model X^2^=27.054, p=0.005, model Nagelkerke R^2^=0.203. Ref. is the reference category against which the odds ratios of the other categories are compared. TNM: tumor, node, metastasis; GEARS: Global Evaluative Assessment of Robotic Skills

Variables	Odds ratio	95% CI	Significance
Overall GEARS score	1.218	0.807-1.836	0.348
Procedure length (min)	1.007	1.004-1.011	<0.001
Sex			
Male	Ref.	Ref.	Ref.
Female	1.323	0.653-2.681	0.436
Age at time of surgery (years)	1.009	0.983-1.036	0.499
BMI at time of surgery (kg/m^2^)	0.988	0.933-1.046	0.682
Neoadjuvant therapy	0.800	0.336-1.905	0.615
History of prior surgery	1.216	0.534-2.772	0.641
TNM stage I	1.075	0.447-2.586	0.872
TNM stage II	0.435	0.152-1.247	0.121
TNM stage III	0.436	0.146-1.306	0.138
TNM stage IV	0.269	0.022-3.273	0.303

The overall GEARS score was further subdivided into its individual components to ascertain if any one component may have a statistically significant association. Mean robotic control score, depth perception, efficiency score, bimanual dexterity score, and force sensitivity score for all procedures were 4.2±0.2, 4.0±0.2, 3.7±0.3, 4.0±0.2, and 4.0±0.2, respectively. Each component was included as a new independent variable in multivariate analysis, and the overall GEARS score was removed. Similarly, no individual component of the GEARS score was found to be significant in predicting the likelihood of postoperative complications (Table 3). However, increased procedure length was again identified as a significant factor in predicting increased odds of postoperative complications (OR=1.007, p<0.001). This model was also significant (X^2^=29.562, p=0.014), with the model accounting for 22% of the variance (Nagelkerke R^2^).

**Table 3:** Multiple logistic regression analysis with overall GEARS score divided into individual subcomponents. Model X^2^=29.562 (p=0.014), model Nagelkerke R^2^=0.220. Ref. is the reference category against which the odds ratios of the other categories are compared. TNM: tumor, node, metastasis; GEARS: Global Evaluative Assessment of Robotic Skills

Variables	Odds ratio	95% CI	Significance
Procedure length (min)	1.007	1.004, 1.011	<0.001
Sex			
Male	Ref.	Ref.	Ref.
Female	0.609	0.586-2.492	0.609
Age at time of surgery (years)	1.010	0.983-1.038	0.474
BMI at time of surgery (kg/m^2^)	0.984	0.929-1.043	0.591
Neoadjuvant therapy	0.801	0.332-1.932	0.622
History of prior surgery	1.147	0.495-2.661	0.749
TNM stage I	1.061	0.435-2.588	0.896
TNM stage II	0.441	0.152-1.283	0.133
TNM stage III	0.425	0.140-1.289	0.131
TNM stage IV	0.267	0.021-3.423	0.310
Robotic control score	3.459	0.320-37.413	0.307
Depth perception score	0.253	0.026-2.470	0.237
Efficiency score	1.948	0.332-11.443	0.461
Bimanual dexterity score	0.684	0.071-6.552	0.742
Force sensitivity score	2.057	0.292-14.499	0.469

The median procedure length was 270 min. The study population was subdivided into long (270 min or more) and short procedures (less than 270 min). Descriptive statistics and clinical data were compiled for each subgroup (Table 1). T-test analysis revealed the mean overall GEARS score was lower in the long procedure group (19.7±0.94) as compared to the short procedure group (20.2±0.7) (p=0.001). There was no significant difference in mean age (p=0.252) or BMI (p=0.125) between the subgroups. In the short procedure group, the total number of patients who experienced at least one postoperative complication was 27 (33.3%, 4.9% C-D grade 3+). Finally, multiple logistic regression analysis was performed for the short procedure subset and revealed that only increased procedure length conferred increased odds of postoperative complications (OR=1.013, p=0.021) (Table 4). However, the model was not shown to be significant (X^2^=8.633, p=0.656). In the long procedure group, the total number of patients who experienced at least one postoperative complication as defined in the methods section was 58 (70%, 19.3% C-D grade 3+). Thus, the long procedure group experienced higher rates of overall postoperative complications as well as higher rates of more serious complications (C-D ≥3) than the short procedure group. Additionally, multiple logistic regression analysis was performed on the long procedure subgroup. The model was shown to be significant (X^2^=19.736, p<0.05) and accounted for 29.3% of variance (Nagelkerke R^2^). Like the previous analyses, overall GEARS score was not identified as a significant predictor of postoperative complications in the long procedure cohort (OR=1.578, p=0.129) (Table 4). Interestingly, procedure length was no longer identified as a significant predictor in the long procedure subgroup (OR=1.002, p=0.539). Pearson correlation was again performed to assess the relationship between overall GEARS score and procedure length; however, no significant relationship was found in either the long (r = -0.162, p=0.144) or short (r = -0.061, p=0.588) procedure subgroups.

**Table 4:** Multiple logistic regression analysis separated by long and short procedures. Model including short procedures only - model X^2^=8.633 (p=0.656), model Nagelkerke R^2^=0.140; model including long procedures only - model X^2^=19.736 (p=0.049), model Nagelkerke R^2^=0.293. Ref. is the reference category against which the odds ratios of the other categories are compared. TNM: tumor, node, metastasis; GEARS: Global Evaluative Assessment of Robotic Skills

Variables	Odds ratio	95% CI	Significance
Short procedures			
Overall GEARS score	0.853	0.416-1.747	0.664
Procedure length (min)	1.013	1.002-1.024	0.021
Sex			
Male	Ref.	Ref.	Ref.
Female	0.570	0.193-1.687	0.310
Age at time of surgery (years)	1.007	0.970-1.046	0.705
BMI at time of surgery (kg/m^2^)	1.006	0.926-1.092	0.893
Neoadjuvant therapy	0.652	0.112-3.805	0.635
History of prior surgery	0.710	0.219-2.297	0.576
TNM stage I	0.618	0.171-2.227	0.462
TNM stage II	0.665	0.139-3.168	0.608
TNM stage III	0.211	0.017-2.642	0.228
TNM stage IV	1.452	0.074-28.378	0.806
Long procedures			
Overall GEARS score	1.578	0.875-2.847	0.129
Procedure length (min)	1.002	0.995-1.009	0.539
Sex			
Male	Ref.	Ref.	Ref.
Female	2.458	0.750-8.054	0.137
Age at time of surgery (years)	1.005	0.958-1.053	0.850
BMI at time of surgery (kg/m^2^)	1.010	0.919-1.111	0.830
Neoadjuvant therapy	1.144	0.360-3.635	0.820
History of prior surgery	1.728	0.441-6.772	0.433
TNM stage I	1.561	0.324-7.524	0.579
TNM stage II	0.212	0.044-1.025	0.054
TNM stage III	0.336	0.072-1.563	0.164
TNM stage IV	0	0-0	0.158

## Discussion

Our results did not confirm a relationship between the overall GEARS score and the odds of experiencing a postoperative complication in robotic-assisted surgical oncology procedures of the UGI and HPB systems. Manual video-based review alone may be inadequate in assessing the quality of surgical technique in this patient population and its potential impact on postoperative outcomes. However, multivariate analysis did reveal a relationship between total operative time and the occurrence of postoperative complications in the overall study population. Specifically, longer operative times were independently associated with increased odds of postoperative complications (OR: 1.007 per min, p<0.001), translating to an approximately 52% increase in odds for each additional hour of operative duration. This trend is consistent with the current literature, which describes operative time as an independent risk factor for postoperative complications in both minimally invasive and traditional surgical approaches across subspecialties [15-18].

One possible explanation for this observation may be in part due to the relationship between longer operative times and decreased surgical experience, implying that longer procedures and, in turn, higher rates of complications may ultimately find their root cause in lower surgical technical skill [19-21]. Support for this notion is demonstrated first by the finding that overall GEARS score was inversely related to operative time for the overall study population. In addition, there was a significant decrease in the average GEARS score between the long- and short-procedure subgroups. Thus, our data clearly demonstrate that those procedures with lower technical skill ratings by the current standard of manual video-based analysis tended to have increased operative times, which further correlated with an overall increase in complications. On the other hand, this finding also suggests that while overall GEARS score may be valuable in assessing some aspects of surgical technique in UGI and HPB surgery, the concurrent finding that the GEARS score, as an independent variable, does not relate to the odds of experiencing a postoperative complications in our study population reveals that the score may not capture the entire picture and its association with postoperative complication risk.

One of these significant surgical technical factors may be inherent procedure complexity. Recent studies regarding newly developed procedure complexity scores have revealed that surgical complexity scores have the potential to better predict postoperative morbidity as compared to more well-established risk assessment measures, including the Charlson Comorbidity Index (CCI), the Elixhauser Comorbidity Index (ECI), and the Centers for Medicare and Medicaid Services Hierarchical Condition Category (CMS-HCC) [22]. This study also revealed that patients with a tumor or liver disease, conditions prevalent within our study population, were among the most complex surgical patients and were highly associated with postoperative complications [22]. In addition, our data revealed higher rates of neoadjuvant chemotherapy, past surgical history, and diagnosed cancer in the long-procedure subgroup as compared to the short-procedure subgroup, suggesting that more advanced disease processes may have resulted in more complex surgical cases that in turn necessitated increased operative times. The observation that more complex procedures generally necessitate longer operative times and result in more postoperative complications at baseline may help explain in part why the GEARS score, which is currently unable to account for this important predictor of surgical outcome, was also unable to predict postsurgical outcomes in our overall study population [22-24].

One potential path forward is incorporating motion tracking devices and data to better quantify surgical performance. Several studies have reported the ability to differentiate surgeon skill level and operative performance on standardized tasks based on the results of motion tracking data alone [25,26]. In addition, motion tracking data have even been used to successfully evaluate surgical resident abilities and provide expert feedback using motion plots to both identify areas of learning needs as well as relevant portions of procedure videos for coaching opportunities [27]. However, while this approach has been successfully implemented in the context of traditional laparoscopic surgery, little data exist concerning the quantification of movements in robotic surgery. Thus, incorporating automated motion tracking data into the current framework of video-based review may have the dual benefit of providing more objective data to better assess surgical performance while also increasing the efficiency of video-based review to develop a performance metric that can more accurately predict patient outcomes.

In addition, further multiple logistic regression analysis of the short-procedure subgroup revealed that only increased procedure length conferred increased odds of postoperative complications (OR=1.013, p=0.021). However, chi-square analysis of the model suggested that our model did not outperform the null in this scenario (X^2^=8.633, p=0.656). Thus, these results, while consistent with our previous findings, must be interpreted with caution. Finally, multiple logistic regression analysis of the long-procedure subgroup identified no significance of any independent covariate. These results imply that above a certain threshold, increases in procedure length begin to have a diminishing effect on postoperative complication odds. Unlike the short-procedure subgroup analysis, the long-procedure subgroup model was verified as significant (X^2^=19.736, p<0.05).

The predominance of upper GI cases in our dataset likely reflects broader procedural volume differences observed in robotic surgery practice. A recent analysis of robotic UGI and HPB procedures demonstrated that robotic UGI operations were performed more frequently than robotic HPB operations, with UGI procedures comprising a larger proportion of robotic general surgery cases [28]. Although robotic HPB surgery continues to expand, many HPB procedures remain concentrated within specialized centers due to technical complexity, resource requirements, and the advanced expertise required for these operations [29].

The major limitation of our study was population size. Inclusion criteria for this study limited our analysis to a smaller portion of the overall dataset available, leading to decreased power and limiting our ability to accurately analyze the effects of other independent variables of interest, including operating surgeon robotic experience level. Moreover, certain subgroup analyses included relatively small numbers of patients within specific disease stages, which may have limited the stability and precision of some regression estimates. Additionally, because only cases with available GEARS assessments were included, the potential for selection bias cannot be excluded. The relatively narrow distribution of GEARS scores within the study population (19.9±0.86) may have also limited our ability to detect associations between technical skill and postoperative outcomes. These limitations were further compounded when the study population was subdivided according to procedure length, leading to some uncertainty in our short procedures model. Finally, formal inter-rater reliability statistics and site-level standardization data were not available for this retrospective cohort, which may limit the reproducibility of the skill assessments.

Future work will be focused upon expanding the existing study population to increase statistical power and allow us to analyze other variables of interest. Additionally, we hope to be able to divide the study population based on hospital site and compare statistics such as mean GEARS score and operative time between our sites to establish patterns, assess potential disparities in outcomes of robotic surgery, and address their root causes. Our team also recognizes that while surgical technical ability may be a prime driver of surgical outcomes, it is far from the only one. Other factors such as preoperative preparation, errors in diagnosis and clinical judgment, patient factors, comorbidities, as well as communication and systems issues, may all affect the postsurgical course. Thus, future research may also incorporate such data for individual patients to place their surgery and postoperative outcomes in a richer, more informative context.

## Conclusions

Manual video-based skill assessments were not associated with postoperative outcomes in this cohort of patients undergoing robotic-assisted UGI and HPB surgery. While increased operative times correlate with lower GEARS scores in the overall study population and have been shown to correlate with lower technical skill, procedure complexity is not accounted for. This has implications for the novice and the expert, who both have a vested interest in accurate reporting of their individual technical skill, especially in the context of high-complexity procedures. Thus, there remains a need for a simple, automated, and objective performance measure that captures technical proficiency throughout the entirety of long procedures while accounting for procedure complexity.

## References

[1] Stulberg JJ, Huang R, Kreutzer L (2020). Association between surgeon technical skills and patient outcomes. JAMA Surg.

[2] Vining CC, Skowron KB, Hogg ME (2021). Robotic gastrointestinal surgery: learning curve, educational programs and outcomes. Updates Surg.

[3] Knab LM, Zureikat AH, Zeh III HJ, Hogg ME (2017). Towards standardized robotic surgery in gastrointestinal oncology. Langenbecks Arch Surg.

[4] Jackson TD, Wannares JJ, Lancaster RT, Rattner DW, Hutter MM (2011). Does speed matter? The impact of operative time on outcome in laparoscopic surgery. Surg Endosc.

[5] Sánchez R, Rodríguez O, Rosciano J, Vegas L, Bond V, Rojas A, Sanchez-Ismayel A (2016). Robotic surgery training: construct validity of Global Evaluative Assessment of Robotic Skills (GEARS). J Robot Surg.

[6] Goh AC, Goldfarb DW, Sander JC, Miles BJ, Dunkin BJ (2012). Global Evaluative Assessment of Robotic Skills: validation of a clinical assessment tool to measure robotic surgical skills. J Urol.

[7] Hogg ME, Zenati M, Novak S (2016). Grading of surgeon technical performance predicts postoperative pancreatic fistula for pancreaticoduodenectomy independent of patient-related variables. Ann Surg.

[8] Goldenberg MG, Goldenberg L, Grantcharov TP (2017). Surgeon performance predicts early continence after robot-assisted radical prostatectomy. J Endourol.

[9] Adam R, Avisar E, Ariche A (2001). Five-year survival following hepatic resection after neoadjuvant therapy for nonresectable colorectal [liver] metastases. Ann Surg Oncol.

[10] Choban PS, Flancbaum L (1997). The impact of obesity on surgical outcomes: a review. J Am Coll Surg.

[11] Ahn KS, Han HS, Yoon YS, Cho JY, Kim JH (2011). Laparoscopic liver resection in patients with a history of upper abdominal surgery. World J Surg.

[12] Perini MV, Montagnini AL, Jukemura J (2008). Clinical and pathologic prognostic factors for curative resection for pancreatic cancer. HPB (Oxford).

[13] Birkmeyer JD, Finks JF, O'Reilly A (2013). Surgical skill and complication rates after bariatric surgery. N Engl J Med.

[14] Clavien PA, Barkun J, de Oliveira ML (2009). The Clavien-Dindo classification of surgical complications: five-year experience. Ann Surg.

[15] Kim BD, Hsu WK, De Oliveira GS, Saha S, Kim JY (2014). Operative duration as an independent risk factor for postoperative complications in single-level lumbar fusion: an analysis of 4588 surgical cases. Spine (Phila Pa 1976).

[16] Manilich E, Vogel JD, Kiran RP, Church JM, Seyidova-Khoshknabi D, Remzi FH (2013). Key factors associated with postoperative complications in patients undergoing colorectal surgery. Dis Colon Rectum.

[17] Scheer A, Martel G, Moloo H, Sabri E, Poulin EC, Mamazza J, Boushey RP (2009). Laparoscopic colon surgery: does operative time matter?. Dis Colon Rectum.

[18] Singh S, Swarer K, Resnick K (2017). Longer operative time is associated with increased post-operative complications in patients undergoing minimally-invasive surgery for endometrial cancer. Gynecol Oncol.

[19] Shigemura K, Yamamichi F, Kitagawa K, Yamashita M, Oka Y, Tanaka H, Fujisawa M (2017). Does surgeon experience affect operative time, adverse events and continence outcomes in holmium laser enucleation of the prostate? a review of more than 1,000 cases. J Urol.

[20] Akman T, Binbay M, Akcay M (2011). Variables that influence operative time during percutaneous nephrolithotomy: an analysis of 1897 cases. J Endourol.

[21] Go CC, Kyin C, Maldonado DR, Domb BG (2020). Surgeon experience in hip arthroscopy affects surgical time, complication rate, and reoperation rate: a systematic review on the learning curve. Arthroscopy.

[22] Hyer JM, White S, Cloyd J, Dillhoff M, Tsung A, Pawlik TM, Ejaz A (2020). Can we improve prediction of adverse surgical outcomes? Development of a surgical complexity score using a novel machine learning technique. J Am Coll Surg.

[23] Jenkins ED, Yom VH, Melman L (2010). Clinical predictors of operative complexity in laparoscopic ventral hernia repair: a prospective study. Surg Endosc.

[24] Reinders JS, Gouma DJ, Heisterkamp J, Tromp E, van Ramshorst B, Boerma D (2013). Laparoscopic cholecystectomy is more difficult after a previous endoscopic retrograde cholangiography. HPB (Oxford).

[25] D'Angelo AL, Rutherford DN, Ray RD (2015). Idle time: an underdeveloped performance metric for assessing surgical skill. Am J Surg.

[26] D'Angelo AL, Rutherford DN, Ray RD, Laufer S, Mason A, Pugh CM (2016). Working volume: validity evidence for a motion-based metric of surgical efficiency. Am J Surg.

[27] Perrone KH, Yang S, Mohamadipanah H, Wise B, Witt A, Goll C, Pugh C (2020). Translating motion tracking data into resident feedback: an opportunity for streamlined video coaching. Am J Surg.

[28] Hur-Thompson E, Gupta S, Duffy S, Chen X, Richardson A, Lau NS, Apostolou C (2026). Trends in robotic upper gastrointestinal and hepatopancreatobiliary surgery in Australia: a private sector-based analysis. J Robot Surg.

[29] Armstrong M, Lu P, Wang J (2024). Advancing minimally invasive hepato-pancreato-biliary surgery: barriers to adoption and equitable access. Surg Endosc.

